# Understanding motivational and demotivational patterns in higher education: psychometric evidence from a Chilean university sample

**DOI:** 10.3389/fpsyg.2026.1825932

**Published:** 2026-09-16

**Authors:** María Díaz Campillay, Héctor Fuentes Castillo

**Affiliations:** Departamento de Industria y Negocios, Universidad de Atacama, Copiapó, Chile

**Keywords:** academic motivation, cross-cultural adaptation, demotivation, factor analysis, higher education, Latin America, psychometric validation, university students

## Abstract

Academic motivation plays an important role in students’ engagement, academic achievement, and persistence in higher education; however, the psychometric structure of motivational measures may vary across educational and cultural contexts. This study evaluated the psychometric performance of the Motivational and Demotivational Patterns in Academic Settings (MDPAS) scale among Chilean university students. Using a quantitative, non-experimental, cross-sectional design, data were collected from 356 undergraduate students enrolled at a public university in northern Chile. Exploratory analyses indicated a complex multidimensional structure. Parallel analysis suggested up to six potentially retainable factors, although support for the sixth factor was marginal. A theoretically informed five-factor exploratory solution accounted for 36.77% of the extracted variance but showed uneven factor definition and only partial correspondence with the original five-dimensional structure. Three factors were sufficiently defined to support subsequent model-based evaluation. A preliminary confirmatory factor analysis, estimated with WLSMV for ordinal indicators, yielded a refined 18-item, three-factor candidate configuration with substantially improved fit, χ^2^(132) = 415.586, *p* <0.001, CFI = 0.911, TLI = 0.897, RMSEA = 0.078, and SRMR = 0.079. Standardized factor loadings ranged from 0.538 to 0.864. Composite reliability was satisfactory for Factors 1 and 2 (CR = 0.892 and 0.776, respectively) and borderline for Factor 3 (CR = 0.689), whereas Average Variance Extracted remained below 0.50 across all factors, indicating incomplete convergent validity. HTMT values ranged from 0.182 to 0.301 and supported empirical differentiation among the retained factors. Associations with selected sociodemographic and academic variables were generally small and selective and were examined using the exploratory 23-item configuration prior to CFA refinement. Overall, the findings provide partial psychometric support for the MDPAS in this Chilean university sample and suggest that a more parsimonious three-factor representation may better characterize the observed motivational and demotivational response structure. Independent replication is required before stronger claims regarding structural stability, cross-cultural equivalence, or broader applied use can be made.

## 1 Introduction

Academic performance and adjustment in higher education are associated with multiple motivational and psychological processes. Among health-sciences university students with a history of low academic performance, personal, psychological, and health-related characteristics have been associated with grade point average, although these findings correspond to a restricted population and should not be generalized to all university students (Brambila-Tapia et al., 2022). Within the motivational domain, meta-analytic evidence indicates that different forms of student motivation are differentially related to performance, persistence, wellbeing, and goal-oriented outcomes (Howard et al., 2021). More autonomous motivational orientations have also been associated with greater satisfaction with studies and certainty about study choice (Puklek Levpušček and Podlesek, 2019), whereas intrinsic motivation and amotivation show contrasting associations with academic procrastination and performance (Arenas Wong et al., 2022).

Self-Determination Theory (SDT) provides one of the most widely applied frameworks for understanding academic motivation. It differentiates forms of motivational regulation according to the degree to which behavior is autonomous or controlled, ranging from intrinsic and self-endorsed forms of motivation to controlled regulation and amotivation (Ryan and Deci, 2020). Studies conducted with university students have documented differentiated patterns of intrinsic motivation, extrinsic regulation, and amotivation, as well as the coexistence of intrinsic and extrinsic motives (Cadête Filho et al., 2021; Vitai and Benke, 2021). Consistent with this distinction, evidence from medical education indicates that relative autonomous motivation is indirectly associated with academic performance through deeper study strategies and greater study effort (Kusurkar et al., 2013).

The satisfaction of the basic psychological needs for autonomy, competence, and relatedness is central to SDT. Meta-analytic evidence indicates that psychological need satisfaction and autonomy support from teachers and parents are associated with more self-determined forms of student motivation, with teacher autonomy support showing stronger associations than parental support (Bureau et al., 2022). Evidence from first-year university students in Malaysia also links the satisfaction of these needs to greater eudaimonic wellbeing (Leow et al., 2023). Systematic reviews further indicate that need-supportive and SDT-based educational conditions are associated with academic engagement and other adaptive educational outcomes (Hutomo and Kurniawati, 2024; Wang et al., 2024).

The multidimensional and context-dependent nature of academic motivation has direct measurement implications. Instruments developed in one educational, linguistic, or cultural setting cannot be assumed to retain the same factorial organization and construct interpretation when applied in another. Cross-cultural validation studies have therefore examined the applicability and factorial validity of academic-motivation measures across different populations, supporting context-specific psychometric evaluation (Caleon et al., 2015; Osei Akoto, 2014).

Academic motivation is also increasingly understood as multidimensional, with adaptive and maladaptive processes potentially co-occurring. Students may simultaneously experience intrinsic interest, externally regulated pressure, perfectionistic tendencies, social comparison, self-regulatory demands, and demotivational experiences. These processes can combine in different ways and relate to distinct patterns of engagement, wellbeing, persistence, and academic performance (Guay, 2022; Litalien et al., 2024). This complexity underscores the value of instruments capable of capturing motivational and demotivational processes beyond broad intrinsic–extrinsic distinctions.

Evidence from Latin American higher education highlights the relevance of motivational and psychosocial processes. Among Argentine university students, specific forms of academic motivation differentially predicted procrastination, which in turn negatively predicted academic achievement (Tisocco and Liporace, 2023). In Chile, dropout risk has been associated with academic and psychosocial factors, including multiple failed courses, depressive symptoms, insomnia, low parental support, and pandemic-related deterioration in academic performance and socioeconomic conditions; however, academic motivation was not directly assessed in this study (Martinez-Libano and Yeomans-Cabrera, 2023). Longitudinal research has also documented changes in academic motivation across university trajectories (Marques et al., 2024). Chilean evidence further indicates that satisfaction of basic psychological needs may influence academic performance indirectly through affect and self-reported motivation (González-Arias et al., 2025). These findings reinforce the importance of examining whether motivational measurement structures developed in other contexts operate similarly among Chilean university students.

This issue is particularly relevant because many academic-motivation instruments were developed in cultural, linguistic, disciplinary, and educational contexts that differ from Latin American higher education. Their factorial organization, item functioning, and construct interpretation may therefore change when transferred across populations (Caleon et al., 2015). Cross-cultural research has shown that the salience and empirical differentiation of motivational dimensions may vary across settings, supporting local psychometric evaluation rather than assuming structural equivalence a priori (Ardeńska et al., 2019; Osei Akoto, 2014). Systematic reviews have also noted limitations in the psychometric evidence available for some commonly used motivational measures across diverse educational contexts (Martinez-Libano and Yeomans-Cabrera, 2023; Sánchez-Bolívar and Martínez-Martínez, 2022).

The Motivational and Demotivational Patterns in Academic Settings (MDPAS) scale offers a complementary framework for assessing academic motivation. Developed by Zeynali et al. (2019) and originally validated among Iranian university students studying English as a foreign language, the instrument comprises 36 Likert-type items organized into five dimensions: motivating demotivation, collective/individual motivation, perfectionist/non-perfectionist motivation, intrinsic/extrinsic motivation, and cooperative/competitive motivation. The original validation reported satisfactory model fit and high internal consistency across the proposed dimensions. This measurement architecture differs from that of the Academic Motivation Scale, which operationalizes SDT through intrinsic motivation, extrinsic motivation, and amotivation (Ardeńska et al., 2019), whereas the MDPAS additionally incorporates perfectionist/non-perfectionist, collective/individual, and cooperative/competitive orientations, together with the distinct construct of motivating demotivation (Zeynali et al., 2019).

Its broader conceptual coverage, however, also raises an important psychometric question when the instrument is applied beyond the population in which it was developed. A five-dimensional structure identified in one linguistic, cultural, and disciplinary setting cannot be assumed to reproduce unchanged in another. Items may redistribute across factors, distinctions among dimensions may weaken, or some factors may fail to achieve adequate empirical definition.

This concern is particularly relevant in Chilean higher education, where academic trajectories, institutional demands, performance expectations, and disciplinary cultures may shape how motivational experiences are expressed and interpreted. Cross-cultural adaptation therefore requires more than linguistic translation; it also requires empirical evaluation of factorial structure, score reliability, and construct-related evidence before structural equivalence can be assumed (Muñiz et al., 2013).

Factor-retention decisions are consequently central to this type of psychometric evaluation. Theoretical structure provides an important comparative framework, but the number and composition of factors identified in a new population must also be supported empirically. Dimensionality, item–factor relationships, factor definition, internal consistency, and model-based fit should therefore be considered jointly when evaluating whether an existing measurement structure transfers adequately across contexts.

Accordingly, the present study aimed to adapt and evaluate the psychometric performance of the MDPAS in a sample of Chilean university students. Specifically, it examined the exploratory factorial organization of the 36-item instrument, internal consistency of the empirically interpretable dimensions, preliminary model-based plausibility of the retained factor configuration, and exploratory relationships between composite factor scores and selected sociodemographic and academic variables. Rather than assuming replication of the original five-factor structure, the study evaluated the extent to which the dimensional organization proposed by Zeynali et al. (2019) could be recovered and meaningfully interpreted in a Chilean higher education context.

Because the exploratory and model-based analyses were conducted in the same sample, the study was designed to provide preliminary psychometric evidence rather than definitive confirmatory validation. Any refined structure was therefore treated as a candidate configuration requiring independent replication.

## 2 Materials and methods

This section describes the methodological approach used to examine the psychometric properties and cross-cultural applicability of the MDPAS in a Chilean higher education context. It presents the study design, participant characteristics, measurement instrument, translation and cultural adaptation procedures, data collection process, statistical analyses, and ethical considerations. The methodological strategy was structured to evaluate the internal organization, reliability, construct-related evidence, and external associations of the instrument while acknowledging the exploratory nature of the adaptation process.

### 2.1 Study design

This study employed a quantitative, non-experimental, cross-sectional design with an instrumental focus to examine the psychometric properties and cross-cultural applicability of the MDPAS among Chilean university students. This design was appropriate for evaluating factorial structure, internal consistency, and construct-related evidence without implying causal relationships.

### 2.2 Participants and sample

The sample comprised 356 undergraduate students enrolled at a public university in northern Chile. Participants were recruited using nonprobability convenience sampling, whereby individuals were drawn from a source readily accessible to the research team (Andrade, 2021). Because convenience samples may not be representative of the target population, and recruitment was limited to a single institution, the findings should be generalized cautiously beyond the institutional context examined.

Sample size adequacy was evaluated in relation to communalities, factor overdetermination, and model complexity rather than solely through fixed participant-to-item ratios (MacCallum et al., 1999). Empirical reviews indicate that samples exceeding 200 participants and participant-to-item ratios above 5:1 are frequently reported in scale-validation studies, although these thresholds should be regarded as descriptive rather than definitive criteria (Anthoine et al., 2014; Kyriazos, 2018). With 356 participants and 36 MDPAS items, the present study yielded a participant-to-item ratio of approximately 9.9:1, supporting the planned exploratory analyses. However, this numerical adequacy does not ensure factorial stability and does not offset the external-validity limitations arising from nonprobability, single-institution recruitment.

Eligibility criteria required participants to be enrolled in an undergraduate program and to have completed at least one academic semester. Participation was voluntary and anonymous. Mean age was 22.9 years (SD = 2.6; range = 19–30). Of the participants, 59.6% identified as female (*n* = 212), 38.2% as male (*n* = 136), and 2.2% did not report gender (*n* = 8). Students were enrolled between the second and twelfth academic semesters, with the highest representation in the second (30.3%) and fourth (19.7%) semesters. Degree programs included Engineering, Health Sciences, Administration, and Social Sciences.

### 2.3 Instruments

The study employed the Motivational and Demotivational Patterns in Academic Settings (MDPAS) scale as the primary instrument for assessing students’ motivational and demotivational patterns. Selected sociodemographic and academic variables were also collected to characterize the sample and explore their relationships with the motivational dimensions identified through the psychometric analyses.

#### 2.3.1 Motivational and Demotivational Patterns in Academic Settings (MDPAS) Scale

Academic motivational and demotivational patterns were assessed using the Motivational and Demotivational Patterns in Academic Settings (MDPAS) Scale developed by Zeynali et al. (2019). The instrument comprises 36 Likert-type items rated on a six-point response scale. The original version proposes five theoretically derived dimensions: motivating demotivation, collective/individual motivation, perfectionist motivation, intrinsic/extrinsic motivation, and cooperative/competitive motivation. The initial validation, conducted with Iranian university students of English as a foreign language, reported satisfactory model fit indices (χ^2^/df = 2.40; RMSEA = 0.059; SRMR = 0.04; CFI, IFI, GFI, and NFI > 0.90) and high internal consistency coefficients (α = 0.81–0.93), providing empirical support for the factorial structure and internal consistency of the original instrument (Zeynali et al., 2019).

#### 2.3.2 Translation and cultural adaptation

The MDPAS scale, originally developed in English, was adapted for use with Chilean university students informed by established guidelines for the cross-cultural adaptation of psychological instruments (Beaton et al., 2000; Hambleton, 2005; Muñiz et al., 2013). The adaptation process prioritized conceptual and semantic equivalence over literal translation. An initial forward translation into Spanish was conducted by bilingual researchers with experience in higher education and educational research. The translated version was subsequently reviewed by a panel of experts in higher education and psychometrics to assess item clarity, cultural relevance, and conceptual consistency, with discrepancies resolved through consensus.

A pilot test involving 20 undergraduate students was then conducted to evaluate item comprehensibility and response clarity. Based on participant feedback, minor lexical adjustments were introduced without altering the original conceptual meaning of the items. Although a formal back-translation procedure was not implemented, expert review and pilot testing were used to assess the semantic comprehensibility and contextual suitability of the Spanish-language version, consistent with the emphasis placed on conceptual and contextual equivalence in cross-cultural instrument adaptation (Hambleton, 2005; Muñiz et al., 2013). Nevertheless, the absence of a formal back-translation procedure limits the extent to which full linguistic equivalence with the original English version can be independently verified. Accordingly, claims regarding linguistic equivalence and cross-cultural comparability were interpreted cautiously.

#### 2.3.3 Participant characteristics and external academic variables

In addition to the 36 MDPAS items, six sociodemographic and academic variables were collected: gender, age, degree program, academic semester, academic performance, and number of failed courses. These variables were treated as external variables and were not considered indicators of the latent motivational constructs assessed by the MDPAS. Accordingly, they were excluded from the exploratory and confirmatory factor analyses.

These variables served two complementary purposes: first, to characterize the study sample and, second, to explore whether motivational and demotivational patterns were associated with selected sociodemographic and academic characteristics. Previous research has documented associations between students’ motivational processes and demographic, academic, and educational characteristics, supporting their inclusion as external variables in the present study (Howard et al., 2021; Puklek Levpušček and Podlesek, 2019).

### 2.4 Procedure and data analysis

Data were collected during regular class sessions after written informed consent had been obtained. Participation was voluntary, anonymous, and uncompensated. The analytical dataset included responses from 356 undergraduate students to the 36 MDPAS items together with six sociodemographic and academic variables: gender, age, degree program, academic semester, academic performance, and number of failed courses.

Data analysis followed a sequential psychometric strategy. Preliminary screening included inspection of response ranges, missing values, potential outliers, and univariate distributional characteristics based on skewness and kurtosis. No missing values were identified in the 36 MDPAS items; therefore, all 356 cases were retained for the psychometric analyses. External-variable analyses used available cases as appropriate; specifically, gender-based comparisons were conducted among the 348 participants who reported either female or male gender.

Psychometric analyses were conducted on the 36 original MDPAS items. Exploratory Factor Analysis (EFA) was performed using Maximum Likelihood extraction with oblique Promax rotation, given the expectation that the underlying motivational dimensions would be correlated (Costello and Osborne, 2005; Lloret-Segura et al., 2014). Factorability was assessed using the Kaiser–Meyer–Olkin (KMO) measure and Bartlett’s test of sphericity.

Factor retention was evaluated using complementary empirical and substantive criteria, including the eigenvalue pattern, scree plot, parallel analysis, communalities, salient loadings, cross-loadings, factor overdetermination, theoretical interpretability, and correspondence with the original MDPAS structure (Goretzko, 2025). Parallel analysis was conducted in IBM SPSS Statistics version 29 using 10,000 datasets generated by independently permuting responses within each item column. This column-wise permutation preserved the marginal distribution of each item while disrupting the observed inter-item correlation structure. The 95th percentile of the resulting random eigenvalue distributions was used as the retention benchmark. Observed eigenvalues exceeding their corresponding random-data benchmarks were considered potentially retainable.

Because the original MDPAS comprises five theoretically defined dimensions, a five-factor EFA was additionally examined as a theoretically informed comparative solution to assess correspondence with the structure proposed by Zeynali et al. (2019), without assuming structural equivalence a priori. Subsequent factor interpretation considered the number and magnitude of salient indicators, cross-loadings, communalities, factor overdetermination, internal consistency, and substantive coherence.

Internal consistency of the exploratory factor configuration was assessed using Cronbach’s alpha and McDonald’s omega. Alpha was retained for comparability with the original MDPAS validation, whereas omega was used as a complementary reliability estimate. Negatively oriented items were reverse-scored using *X*_*R*_ = 7 − *X*, where *X* represents the original response and *X_R_* the reverse-scored value, before reliability estimation and score construction. Composite factor scores were then calculated as the arithmetic mean of the items assigned to each factor in the 23-item exploratory configuration. Higher scores represented greater expression of the corresponding motivational pattern.

Exploratory relationships with external variables were examined using composite factor scores derived from the pre-refinement 23-item exploratory configuration. Spearman’s rank-order correlations were used to examine associations with age, academic semester, academic performance, and number of failed courses. Degree programs were grouped into four academic areas: Engineering and Technology, Health Sciences, Administration, and Social Sciences. Gender differences were examined using Mann–Whitney U tests, whereas academic-area differences were evaluated using Kruskal–Wallis tests followed, when significant, by Bonferroni-adjusted pairwise comparisons. *P*-values from the remaining external-variable analyses were not adjusted for multiple testing and were therefore interpreted cautiously. These analyses were not used to guide factor retention, item removal, or CFA refinement.

Academic performance was operationalized as an ordinal three-category variable based on cumulative grade averages on the Chilean 1.0–7.0 grading scale: 1 = low (4.0–4.9), 2 = medium (5.0–5.9), and 3 = high (6.0–7.0).

A Confirmatory Factor Analysis (CFA) was subsequently conducted to evaluate the fit and plausibility of the exploratory factor configuration. Because the six-point MDPAS responses were treated as ordered categorical indicators, model parameters were estimated using the robust Weighted Least Squares Mean and Variance adjusted estimator (WLSMV), an approach appropriate for CFA with ordinal indicators (Flora and Curran, 2004; Li, 2016). Analyses were conducted in R version 4.6.1 using the lavaan package (Rosseel, 2012).

Model fit was evaluated jointly using the chi-square statistic, Comparative Fit Index (CFI), Tucker–Lewis Index (TLI), Root Mean Square Error of Approximation (RMSEA) with its 90% confidence interval, and Standardized Root Mean Square Residual (SRMR) (Schermelleh-Engel et al., 2003). Standardized factor loadings and modification indices were also inspected to identify localized model misfit. Model refinement considered converging evidence from comparatively weak loadings, cross-factor ambiguity identified in the exploratory analyses, and localized diagnostics rather than global fit improvement alone.

Because the EFA and CFA were conducted using the same sample, the CFA was interpreted as a preliminary model-based assessment rather than as an independent confirmation of the measurement structure. Sequential sensitivity analyses were used to examine indicators showing weak loadings, cross-factor ambiguity, or localized model misfit, and any refined configuration was regarded as provisional pending replication in an independent sample.

Construct-related evidence for the refined CFA configuration was evaluated using composite reliability (CR), average variance extracted (AVE), McDonald’s omega, and the Heterotrait–Monotrait ratio of correlations (HTMT). CR and AVE were examined as indicators of construct reliability and convergent validity, respectively (Fornell and Larcker, 1981), whereas McDonald’s omega was estimated as a complementary reliability coefficient (Dunn et al., 2014). HTMT was used to assess discriminant differentiation (Henseler et al., 2015). These indices were interpreted jointly with standardized factor loadings and global model-fit evidence.

Given the cross-sectional and non-experimental design, observed associations, between-group differences, and model-based relationships were not interpreted causally.

Statistical screening, descriptive analyses, EFA, parallel analysis, exploratory reliability analyses, and non-parametric analyses involving external variables were conducted using IBM SPSS Statistics version 29. CFA and the associated model-based psychometric analyses were conducted in R version 4.6.1 using the lavaan package with WLSMV estimation for ordered categorical indicators. Graphical representations were generated using Python version 3.11.

### 2.5 Ethical considerations

Participation in the study was voluntary and anonymous. Prior to data collection, participants were informed about the purpose of the research, the voluntary nature of their participation, the confidentiality of their responses, and their right to withdraw from the study. Written informed consent was obtained from all participants.

The study followed a non-experimental, cross-sectional design based on anonymous survey data collected from adult university students. No experimental intervention or biological sample collection was involved, and no direct personal identifiers were collected. The data were analyzed in anonymized form and used exclusively for research purposes, with measures taken to preserve participant confidentiality and data protection.

The study procedures were conducted in accordance with the ethical principles of the Declaration of Helsinki, particularly those concerning voluntary participation, informed consent, confidentiality, and protection of participants.

## 3 Results

This section presents the empirical findings obtained from the psychometric evaluation of the MDPAS in the Chilean university sample. The results are organized sequentially, beginning with the exploratory examination of the factorial structure, followed by the assessment of item–factor relationships and internal consistency. Subsequently, associations with selected external sociodemographic and academic variables are examined, and the retained factorial solution is evaluated through a preliminary confirmatory analysis.

### 3.1 Exploratory factor analysis

An Exploratory factor analysis (EFA) was conducted on the 36 MDPAS items using Maximum Likelihood (ML) extraction with oblique Promax rotation. The data were suitable for factor analysis, as indicated by a Kaiser–Meyer–Olkin value of 0.792 and a statistically significant Bartlett’s test of sphericity, χ^2^(630) = 4544.203, *p* < 0.001 (Costello and Osborne, 2005; Lloret-Segura et al., 2014).

Item communalities showed substantial variation across the 36 items (Table 1). The highest values were observed for Items 3 (*h*^2^ = 0.710), 28 (*h*^2^ = 0.673), and 34 (*h*^2^ = 0.633), whereas Items 14 (*h*^2^ = 0.114), 29 (*h*^2^ = 0.115), and 21 (*h*^2^ = 0.163) showed particularly low communalities. Several additional items fell below the conventional 0.30 reference value, indicating limited representation by the common-factor solution (Hair et al., 2019).

**TABLE 1:** Extracted communalities for the 36 MDPAS items in the five-factor EFA solution.

Item	Item summary	Extracted communality (h^2^)
1	Professors’ incompetence motivates greater effort	0.230
2	Academic expectations are realistic	0.469
3	Enjoy studying the degree program	0.710
4	Does not compare oneself with others	0.434
5	Enjoys learning through materials and books	0.305
6	Making friendships in class is important	0.270
7	Does not need others for encouragement	0.382
8	Studies to obtain a better job	0.283
9	Does not become discouraged after a poor grade	0.263
10	Complete mastery of degree subjects is a goal	0.225
11	Degree title and prestige are important	0.272
12	Negative comments affect motivation	0.413
13	Does not need to be the most successful among friends	0.271
14	Studies to earn more money	0.114
15	Being the best in the field is important	0.395
16	No matter how much one studies, it is never enough	0.349
17	Does not worry about others’ opinions	0.462
18	Studying the degree broadens one’s worldview	0.520
19	Wants to complete the education already started	0.389
20	Does not care about others’ opinions of the degree	0.314
21	Poor university facilities motivate greater effort	0.163
22	Obtaining a good grade is very important	0.400
23	Wants to demonstrate abilities to others	0.397
24	Motivation is influenced by classmates	0.347
25	Searches for better material for self-learning	0.308
26	Performs better in friendly, non-competitive environments	0.356
27	Values learning for its own sake	0.525
28	Studying the degree aligns with personal goals	0.673
29	Prefers group activities and cooperation	0.115
30	Competitive labor market motivates being the best	0.466
31	Holds high expectations for oneself in education	0.351
32	Was not influenced by others when choosing the degree	0.333
33	Competition with classmates promotes improvement	0.411
34	Chose the degree because of interest in the discipline	0.633
35	Attends university to demonstrate success to family	0.408
36	Negative social attitudes toward the degree motivate greater effort	0.282

Communalities represent the proportion of each item’s variance accounted for by the common factors. Values below 0.30 indicate comparatively weak common variance (Hair et al., 2019).

Kaiser’s eigenvalue-greater-than-one criterion identified 11 factors, accounting for 64.79% of the total variance based on the initial eigenvalues. Given the tendency of this criterion to overextract factors, factor retention was further examined using parallel analysis and substantive criteria.

Parallel analysis was based on 10,000 datasets generated through independent column-wise permutations of the observed item responses, using the 95th percentile of the random eigenvalue distributions as the reference criterion. The first six observed eigenvalues exceeded their corresponding random-data benchmarks. However, support for the sixth factor was marginal: its observed eigenvalue was 1.418 compared with a 95th-percentile random eigenvalue of 1.410. The seventh observed eigenvalue (1.225) fell below its corresponding random benchmark (1.367). Parallel analysis therefore suggested up to six potentially retainable factors, although evidence for the sixth was weak.

Inspection of the six-factor solution showed that the additional factor was essentially defined by a single salient indicator and lacked sufficient empirical overdetermination and substantive interpretability. Accordingly, the marginal sixth factor was interpreted as evidence of additional but poorly defined structural complexity rather than as support for a stable sixth latent dimension; therefore, the six-factor solution was not retained. Factor retention was instead evaluated by jointly considering the parallel-analysis results, scree plot, communalities, rotated pattern coefficients, cross-loadings, factor definition, theoretical interpretability, and correspondence with the original MDPAS structure (Costello and Osborne, 2005; Lloret-Segura et al., 2014).

Because the original MDPAS proposes five theoretical dimensions (Zeynali et al., 2019), a five-factor EFA was examined as a theoretically informed comparative solution rather than as an assumption of structural equivalence. This solution accounted for 36.77% of the variance extracted by the common factors: 17.17% for Factor 1, 8.22% for Factor 2, 4.92% for Factor 3, 3.64% for Factor 4, and 2.82% for Factor 5 (Table 2).

**TABLE 2:** Variance accounted for by the five-factor Maximum Likelihood solution.

Factor	Initial eigenvalue	Initial variance (%)	Cumulative initial variance (%)	Extracted variance (%)	Cumulative extracted variance (%)
1	6.789	18.86	18.86	17.17	17.17
2	3.578	9.94	28.80	8.22	25.39
3	2.399	6.66	35.46	4.92	30.31
4	1.896	5.27	40.73	3.64	33.95
5	1.652	4.59	45.32	2.82	36.77

The five-factor solution was specified for exploratory comparison with the theoretical structure proposed by Zeynali et al. (2019). Because an oblique Promax rotation was used, rotated sums of squared loadings were not interpreted as additive percentages of explained variance.

The scree plot showed a pronounced decline across the first factors followed by a progressively flatter trajectory (Figure 1). Examination of the five-factor rotated solution indicated uneven recovery of the original dimensions: some item clusters were comparatively coherent, whereas others showed weak primary loadings, cross-factor redistribution, or insufficient factorial definition. The item-level structure and the basis for retaining the sufficiently defined dimensions are reported in section 3.2.

**FIGURE 1:**
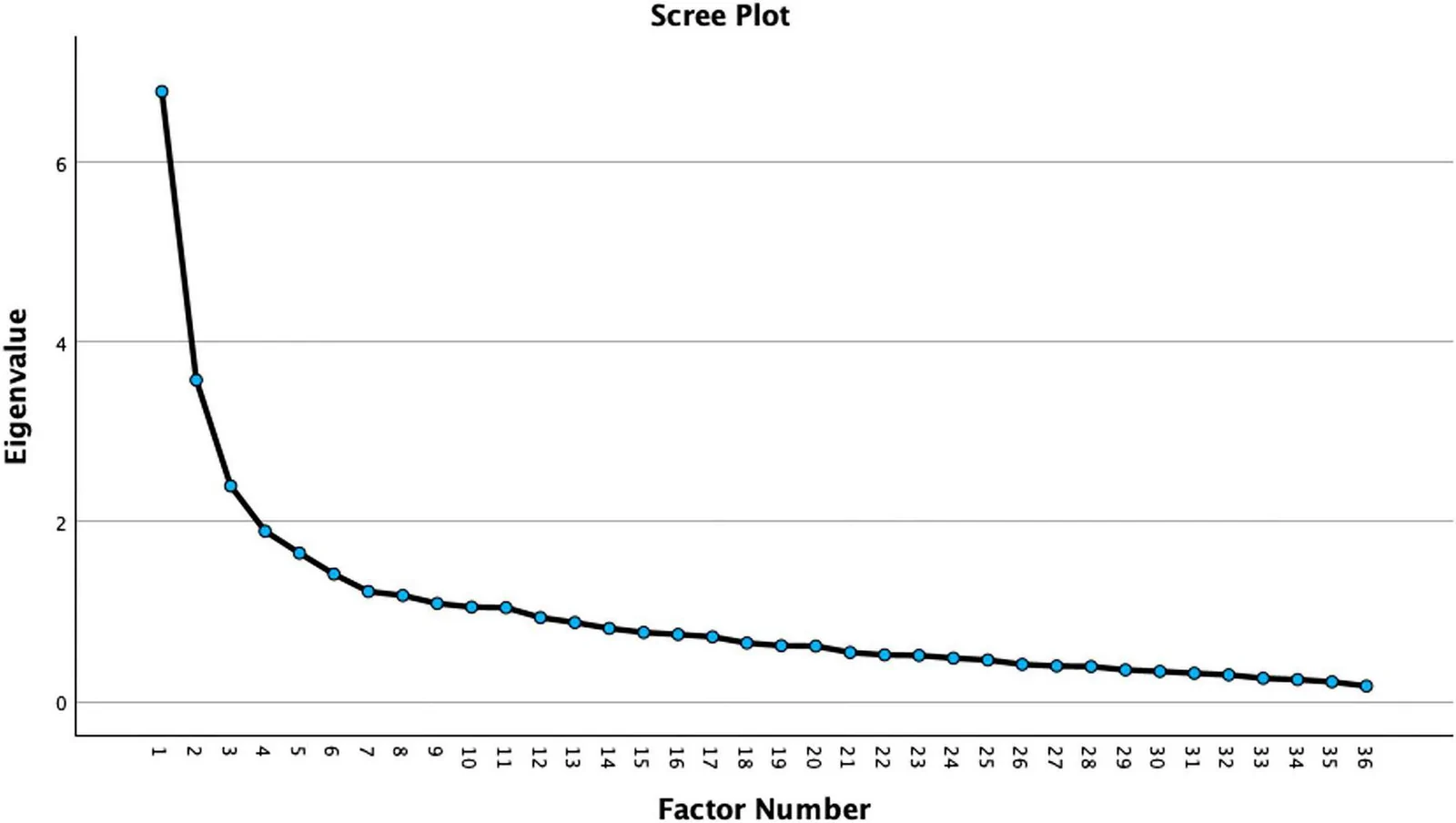
Scree plot of initial eigenvalues for the 36 MDPAS items. The scree plot was interpreted jointly with the parallel-analysis results and the item-level characteristics of the rotated solution rather than as an independent factor-retention criterion.

### 3.2 Factor structure and item-level examination

The Promax-rotated pattern matrix of the theoretically informed five-factor solution was examined to characterize item–factor relationships. Because Promax allows correlations among factors, the pattern matrix was used as the primary basis for interpretation. Pattern coefficients with absolute values ≥ 0.40 were considered salient for descriptive purposes (Hair et al., 2019), although retention decisions also considered cross-loadings, communalities, factor overdetermination, theoretical coherence, and the number of salient indicators defining each factor (Costello and Osborne, 2005; Lloret-Segura et al., 2014).

The five-factor solution showed uneven correspondence with the original MDPAS structure proposed by Zeynali et al. (2019). Factor 1 was the most clearly defined dimension, with ten salient positive loadings ranging from 0.427 to 0.831. The strongest indicators were Items 28 (λ = 0.831), 18 (λ = 0.778), 34 (λ = 0.732), 19 (λ = 0.699), 3 (λ = 0.648), and 27 (λ = 0.622). Although most reflected the original intrinsic/extrinsic motivation dimension, Items 31, 25, 23, and 5 originated from other dimensions, indicating cross-dimensional redistribution.

Factor 2 comprised eight salient indicators, with absolute loadings ranging from 0.429 to 0.639. Its strongest indicators were Items 7 (λ = 0.639), 17 (λ = 0.634), 12 (λ = −0.632), 4 (λ = 0.623), 9 (λ = 0.494), and 20 (λ = 0.458), with Items 2 (λ = 0.436) and 16 (λ = −0.429) also loading saliently. The factor combined indicators originally assigned to collective/individual motivation with content from the perfectionist/non-perfectionist and cooperative/competitive dimensions, indicating only partial correspondence with the original structure.

Factor 3 contained five salient indicators and showed a comparatively coherent competitive and performance-oriented pattern: Item 30 (λ = 0.601), Item 26 (λ = −0.562), Item 33 (λ = 0.508), Item 13 (λ = −0.499), and Item 15 (λ = 0.487). This factor combined content from the original cooperative/competitive and perfectionist/non-perfectionist dimensions rather than reproducing either dimension independently.

Factors 4 and 5 were weakly defined. Factor 4 contained only Items 35 (λ = 0.483) and 22 (λ = 0.416), whereas Factor 5 included one unique salient indicator, Item 6 (λ = 0.486), together with Item 35 (λ = 0.464), which cross-loaded on Factor 4. The limited number of unique indicators and the cross-loading of Item 35 indicated insufficient factor overdetermination for both dimensions.

Ten items did not reach the | 0.40| criterion on any factor: Items 1, 8, 10, 11, 14, 21, 24, 29, 32, and 36. Several also showed low communalities, particularly Items 14 and 29. Overall, the item-level pattern indicated only partial recovery of the original five-dimensional MDPAS structure.

Table 3 summarizes the salient pattern coefficients and their correspondence with the original theoretical dimensions.

**TABLE 3:** Salient pattern coefficients in the five-factor Promax solution.

Empirical factor	Salient items and pattern coefficients	Predominant theoretical content	Main psychometric issue
Factor 1	28 (0.831), 18 (0.778), 34 (0.732), 19 (0.699), 3 (0.648), 27 (0.622), 31 (0.515), 25 (0.484), 23 (0.433), 5 (0.427)	Predominantly intrinsic/extrinsic motivation, with indicators from other original dimensions	Cross-dimensional redistribution of items
Factor 2	7 (0.639), 17 (0.634), 12 (−0.632), 4 (0.623), 9 (0.494), 20 (0.458), 2 (0.436), 16 (−0.429)	Collective/individual orientation with additional perfectionist and cooperative/competitive content	Partial correspondence with original dimension
Factor 3	30 (0.601), 26 (−0.562), 33 (0.508), 13 (−0.499), 15 (0.487)	Competitive/performance-oriented motivation	Combination of two original dimensions
Factor 4	35 (0.483), 22 (0.416)	External/social achievement orientation	Only two salient indicators
Factor 5	6 (0.486), 35 (0.464)	Social/cooperative orientation	Only one unique salient indicator; Item 35 cross-loads with Factor 4

Only pattern coefficients with absolute values ≥ 0.40 are displayed. Item 35 showed salient loadings on both Factor 4 (0.483) and Factor 5 (0.464). Items 1, 8, 10, 11, 14, 21, 24, 29, 32, and 36 did not reach the | 0.40| criterion on any factor. Extraction method: Maximum Likelihood; rotation method: Promax with Kaiser normalization.

To complement the numerical results, the pattern matrix was visualized as a heat map (Figure 2), highlighting item clustering and cross-factor dispersion.

**FIGURE 2:**
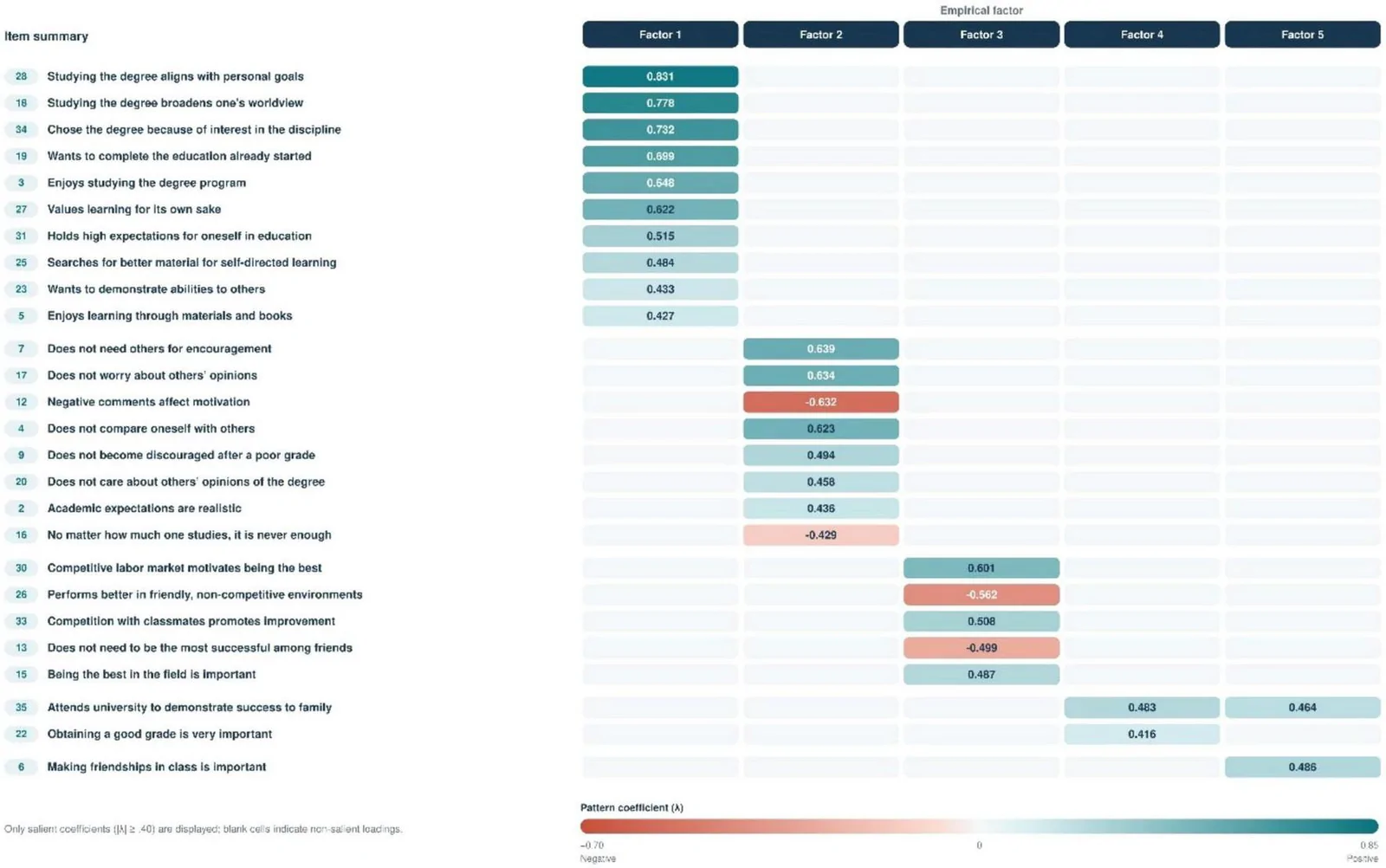
Heat map of pattern coefficients from the five-factor Promax solution. Pattern coefficients were obtained from the Maximum Likelihood exploratory factor analysis with Promax rotation. The heat map displays only items with at least one pattern coefficient with an absolute value ≥ 0.40 across the five specified factors. Positive and negative coefficients indicate the direction of the association between each item and factor.

Based on factor definition, indicator coverage, and interpretability, only Factors 1–3 were considered sufficiently stable for subsequent model-based evaluation. These factors comprised 23 indicators: 10 for Factor 1, eight for Factor 2, and five for Factor 3. Factors 4 and 5 were excluded because of insufficient numbers of unique salient indicators, cross-loading, and weak factor overdetermination (Costello and Osborne, 2005; Hair et al., 2019). The resulting 23-item, three-factor configuration was therefore carried forward to the CFA as an empirically informed reduction of the exploratory solution rather than as confirmation of the original five-dimensional MDPAS structure.

### 3.3 Internal consistency

Internal consistency was assessed using Cronbach’s alpha and McDonald’s omega. Alpha was retained for comparability with the original MDPAS validation, whereas omega was used as a complementary reliability estimate. Coefficients around 0.70 were considered broadly acceptable for research purposes, with lower values interpreted cautiously in relation to factor definition and indicator coverage (Nunnally and Bernstein, 1994; Tavakol and Dennick, 2011).

For the full 36-item instrument, Cronbach’s alpha was approximately 0.81. Given the substantial redistribution of items observed in the exploratory analyses, this coefficient was treated as descriptive rather than as evidence of unidimensionality or replication of the original MDPAS structure. Reliability was therefore examined separately for the empirically interpretable factors retained from the five-factor solution.

Before factor-specific reliability estimation, Items 12 and 16 in Factor 2 and Items 26 and 13 in Factor 3 were reverse-scored to align their direction with the remaining indicators.

Factor 1 showed good internal consistency (α = 0.858; ω = 0.860), and Factor 2 showed acceptable consistency (α = 0.762; ω = 0.756). Factor 3 yielded lower coefficients (α = 0.668; ω = 0.667), indicating borderline to moderate reliability. By contrast, Factors 4 and 5 showed weak internal consistency (α = 0.330 and 0.426, respectively) and were also poorly defined by their indicator structure (Table 4). Given their limited number of unique salient indicators and the cross-loading of Item 35, these factors were not interpreted as stable subscales (Nunnally and Bernstein, 1994; Tavakol and Dennick, 2011).

**TABLE 4:** Internal consistency coefficients for the exploratory empirical MDPAS factor structure.

Factor	Retained items	n items	Cronbach’s α	McDonald’s ω	Interpretation
Factor 1	28, 18, 34, 19, 3, 27, 31, 25, 23, 5	10	0.858	0.860	Good
Factor 2	7, 17, 12R, 4, 9, 20, 2, 16R	8	0.762	0.756	Acceptable
Factor 3	30, 26R, 33, 13R, 15	5	0.668	0.667	Borderline/moderate
Factor 4	35, 22	2	0.330	–	Weakly defined
Factor 5	6, 35	2*	0.426	–	Weakly defined

R denotes reverse-scored items. Reverse scoring was performed using for the six-point response scale. Factors 4 and 5 were not interpreted as stable subscales because they were defined by an insufficient number of unique salient indicators and showed low internal consistency. Item 35 cross-loaded on Factors 4 and 5. The reliability coefficients reported in this table correspond to the exploratory factor configuration and should be distinguished from the model-based reliability estimates subsequently obtained for the refined 18-item CFA structure. The asterisk (*) indicates that Factor 5 contains two salient items; however, only one is a unique salient indicator because Item 35 also cross-loads on Factor 4.

The exploratory reliability results therefore supported carrying forward Factors 1–3 to the initial CFA, while providing no support for treating Factors 4 and 5 as stable dimensions. These estimates refer to the 23-item exploratory configuration; reliability indices for the refined 18-item candidate model are reported separately in Section 3.5.

### 3.4 Relations with external sociodemographic and academic variables

Associations between the three composite factor scores derived from the 23-item exploratory configuration and selected sociodemographic and academic variables were examined prior to CFA refinement. These analyses were exploratory and were not used to determine factor retention, item removal, or model refinement. Accordingly, they should not be interpreted as external validation of the final 18-item candidate configuration.

Spearman’s rank-order correlations were used to examine associations with age, academic semester, academic performance, and number of failed courses. Academic performance was coded ordinally as 1 = low (4.0–4.9), 2 = medium (5.0–5.9), and 3 = high (6.0–7.0). The Factor 1 composite score was positively associated with age (ρ = 0.198, *p* < 0.001) and academic performance (ρ = 0.281, *p* < 0.001), but not with academic semester or failed courses. The Factor 2 composite score was positively associated with age (ρ = 0.267, *p* < 0.001) and academic performance (ρ = 0.210, *p* < 0.001), and negatively associated with academic semester (ρ = −0.247, *p* < 0.001), while showing no significant association with failed courses. The Factor 3 composite score was negatively associated with academic semester (ρ = −0.137, *p* = 0.009) and positively associated with academic performance (ρ = 0.123, *p* = 0.020); associations with age and failed courses were not statistically significant.

Gender differences were examined using Mann–Whitney U tests among the 348 participants who reported female or male gender. Significant differences were observed for the Factor 1 composite score (*U* = 11,575.00, *Z* = −3.107, *p* = 0.002, *r* = 0.17) and the Factor 3 composite score (*U* = 11,286.00, *Z* = −3.426, *p* < 0.001, *r* = 0.18), whereas the Factor 2 composite score showed no significant difference (*U* = 14,315.00, *Z* = −0.110, *p* = 0.912, *r* = 0.01). Female participants showed higher mean ranks on Factor 1, whereas male participants showed higher mean ranks on Factor 3. Effect sizes were small.

Academic-area differences were examined across Engineering and Technology (*n* = 100), Health Sciences (*n* = 98), Administration (*n* = 116), and Social Sciences (*n* = 42). Kruskal–Wallis tests were statistically significant for Factor 1, *H*(3) = 10.009, *p* = 0.018; Factor 2, *H*(3) = 71.520, *p* < 0.001; and Factor 3, *H*(3) = 11.395, *p* = 0.010 (Table 5). Bonferroni-adjusted pairwise comparisons showed a significant difference between Engineering and Technology and Administration for Factor 1 (adjusted *p* = 0.036); for Factor 2, Administration differed from Engineering and Technology, Health Sciences, and Social Sciences (all adjusted *p* < 0.001); and for Factor 3, Engineering and Technology differed from Health Sciences (adjusted *p* = 0.006). No other pairwise comparisons remained statistically significant after adjustment (Table 6).

**TABLE 5:** Associations between the empirically interpretable MDPAS factors and external sociodemographic and academic variables.

External variable	Factor	Statistical test	Statistic	*p*	Interpretation
Age	F1	Spearman’s correlation	ρ = 0.198	< 0.001	Positive association
	F2	Spearman’s correlation	ρ = 0.267	< 0.001	Positive association
	F3	Spearman’s correlation	ρ = 0.021	0.697	Not significant
Academic semester	F1	Spearman’s correlation	ρ = −0.009	0.868	Not significant
	F2	Spearman’s correlation	ρ = −0.247	< 0.001	Negative association
	F3	Spearman’s correlation	ρ = −0.137	0.009	Negative association
Academic performance	F1	Spearman’s correlation	ρ = 0.281	< 0.001	Positive association
	F2	Spearman’s correlation	ρ = 0.210	< 0.001	Positive association
	F3	Spearman’s correlation	ρ = 0.123	0.020	Positive association
Failed courses	F1	Spearman’s correlation	ρ = −0.081	0.125	Not significant
	F2	Spearman’s correlation	ρ = 0.012	0.827	Not significant
	F3	Spearman’s correlation	ρ = 0.077	0.147	Not significant
Gender	F1	Mann–Whitney U	*U* = 11,575.00; *Z* = −3.107; *r* = 0.17	0.002	Female > male
	F2	Mann–Whitney U	*U* = 14,315.00; *Z* = −0.110; *r* = 0.01	0.912	Not significant
	F3	Mann–Whitney U	*U* = 11,286.00; *Z* = −3.426; *r* = 0.18	< 0.001	Male > female
Academic area	F1	Kruskal–Wallis	H(3) = 10.009	0.018	Significant group differences
	F2	Kruskal–Wallis	H(3) = 71.520	< 0.001	Significant group differences
	F3	Kruskal–Wallis	H(3) = 11.395	0.010	Significant group differences

Spearman’s rank-order correlation was used for age, academic semester, academic performance, and number of failed courses. Academic performance was coded ordinally as 1 = low (4.0–4.9), 2 = medium (5.0–5.9), and 3 = high (6.0–7.0), based on cumulative grade averages on the Chilean 1.0–7.0 grading scale. Gender differences were examined using Mann–Whitney U tests; effect sizes were calculated as *r = | Z| /N*, where | Z| is the absolute standardized test statistic and N is the number of participants included in the comparison. Academic-area differences were evaluated using Kruskal–Wallis tests. Factors 1–3 correspond to the three empirically interpretable factors identified in the 23-item exploratory configuration prior to CFA refinement.

**TABLE 6:** Bonferroni-adjusted pairwise comparisons across academic areas.

Factor	Significant pairwise comparison	Adjusted *p*
F1	Engineering and technology vs. administration	0.036
F2	Engineering and technology vs. administration	< 0.001
F2	Health sciences vs. administration	< 0.001
F2	Administration vs. social sciences	< 0.001
F3	Engineering and technology vs. health sciences	0.006

Only pairwise comparisons that remained statistically significant after Bonferroni adjustment are reported. The four academic areas were Engineering and Technology, Health Sciences, Administration, and Social Sciences.

Overall, the external-variable analyses showed selective and generally small associations. Academic performance was positively related to all three exploratory composite scores, age to the Factor 1 and Factor 2 scores, and academic semester negatively to the Factor 2 and Factor 3 scores; failed courses were unrelated to all three scores. Gender differences were limited to Factors 1 and 3 and showed small effect sizes, while academic-area differences were most pronounced for Factor 2. Because these analyses were exploratory, based on the pre-refinement 23-item configuration, and not corrected globally for multiple testing, they should be interpreted primarily in terms of effect magnitude and pattern rather than statistical significance alone. Given the cross-sectional, non-experimental design, no causal interpretation is warranted.

### 3.5 Confirmatory factor analysis and construct-related evidence

A confirmatory factor analysis (CFA) was conducted as a preliminary model-based evaluation of the 23-item, three-factor configuration retained from the exploratory analyses. Because the EFA and CFA were conducted in the same sample, the CFA was interpreted as preliminary structural evidence rather than as independent confirmation of the measurement model.

Given the six-point ordinal response format, all indicators were treated as ordered categorical variables and estimated using robust Weighted Least Squares Mean and Variance adjusted estimation (WLSMV). Model fit was evaluated using χ^2^, CFI, TLI, RMSEA with 90% confidence intervals, and SRMR. Standardized factor loadings and modification indices were also inspected. Model refinement was based on converging evidence from weak loadings, cross-factor ambiguity identified in the exploratory analyses, and localized model diagnostics rather than on global fit improvement alone.

The initial 23-item, three-factor model showed inadequate fit, χ^2^(227) = 1199.270, *p* < 0.001, CFI = 0.750, TLI = 0.722, RMSEA = 0.110, and SRMR = 0.118. Items 26 and 13 showed comparatively weak loadings on Factor 3 (λ = 0.299 and 0.389, respectively), and their removal improved fit to χ^2^(186) = 874.808, *p* < 0.001, CFI = 0.819, TLI = 0.795, RMSEA = 0.102, and SRMR = 0.104. Item 2 was subsequently removed because of cross-factor ambiguity, yielding χ^2^(167) = 627.417, *p* < 0.001, CFI = 0.870, TLI = 0.852, RMSEA = 0.088, 90% CI (0.081, 0.096), and SRMR = 0.092. Removal of Item 23, which also showed substantial cross-factor ambiguity, further improved fit to χ^2^(149) = 531.542, *p* < 0.001, CFI = 0.884, TLI = 0.867, RMSEA = 0.085, 90% CI (0.077, 0.093), and SRMR = 0.088. Finally, Item 20 was removed because of residual cross-factor misfit. The resulting 18-item, three-factor model showed the best overall fit: χ^2^(132) = 415.586, *p* < 0.001, CFI = 0.911, TLI = 0.897, RMSEA = 0.078, 90% CI (0.069, 0.086), and SRMR = 0.079 (Table 7). Given the sequential within-sample refinement, this specification was treated as a candidate model rather than as a definitive confirmatory solution.

**TABLE 7:** Fit indices across the sequential three-factor CFA specifications.

Model	Model modification	χ ^2^	df	CFI	TLI	RMSEA	SRMR
Initial three-factor model	23 indicators	1199.270	227	0.750	0.722	0.110	0.118
Model 2	Items 26 and 13 removed	874.808	186	0.819	0.795	0.102	0.104
Model 3	Item 2 additionally removed	627.417	167	0.870	0.852	0.088	0.092
Model 4	Item 23 additionally removed	531.542	149	0.884	0.867	0.085	0.088
Final model	Item 20 additionally removed	415.586	132	0.911	0.897	0.078	0.079

CFA models were estimated using WLSMV with indicators treated as ordered categorical variables. All chi-square tests were statistically significant at *p* < 0.001. Model refinement was based on converging evidence from standardized loadings, exploratory factor patterns, cross-factor ambiguity, and localized model diagnostics rather than on global fit indices alone.

The final candidate model comprised 18 indicators across three correlated latent factors. Factor 1 included Items 28, 18, 34, 19, 3, 27, 31, 25, and 5; Factor 2 included Items 7, 17, 12R, 4, 9, and 16R; and Factor 3 included Items 30, 33, and 15. All standardized loadings were statistically significant at *p* < 0.001, ranging from 0.538 to 0.864 for Factor 1, 0.559 to 0.729 for Factor 2, and 0.575 to 0.722 for Factor 3. Latent-factor correlations were modest: *r* = 0.199 between Factors 1 and 2, *r* = 0.360 between Factors 1 and 3, and *r* = −0.037 between Factors 2 and 3 (Figure 3).

**FIGURE 3:**
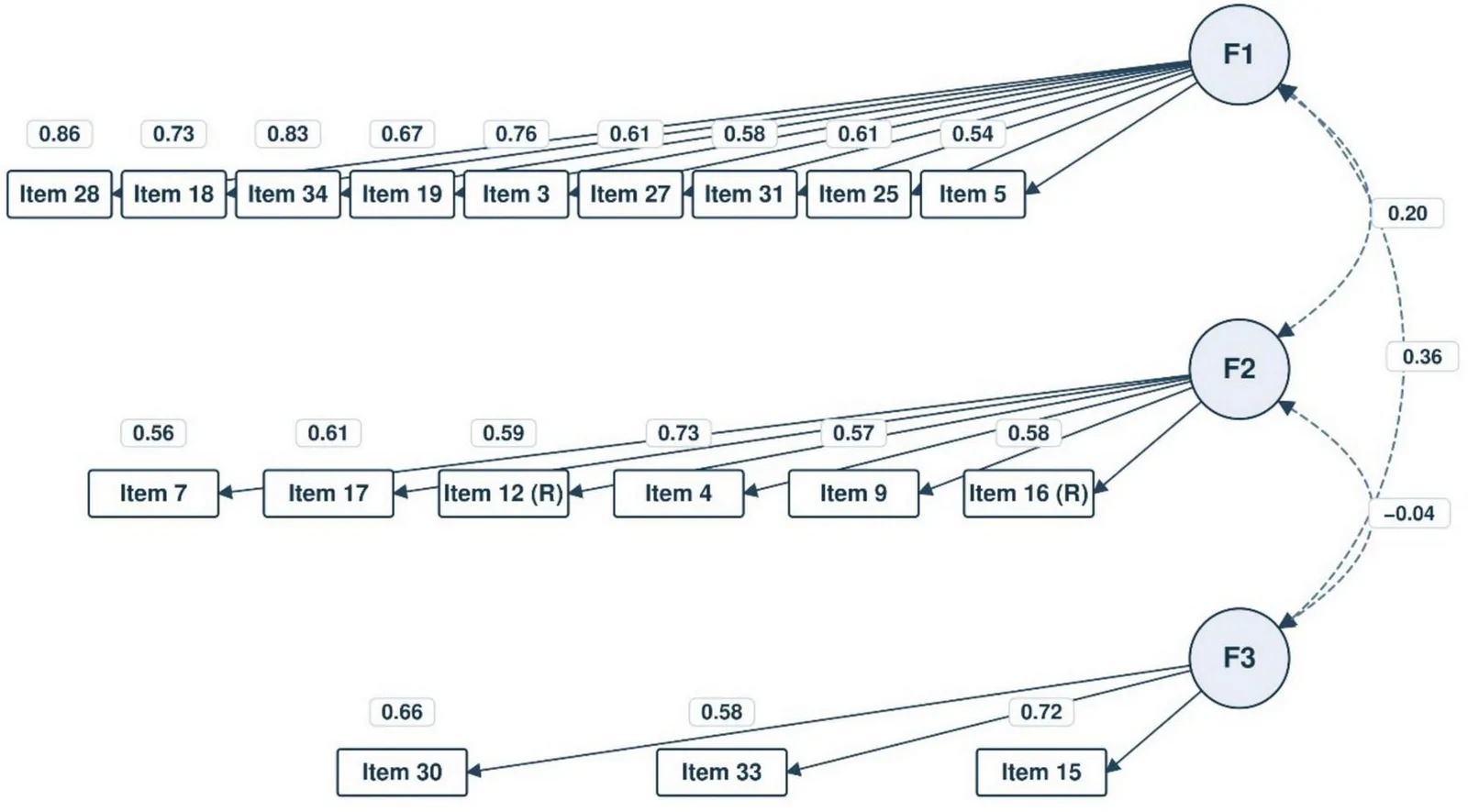
Final three-factor confirmatory measurement model of the MDPAS-derived candidate structure. The figure presents the final 18-item, three-factor candidate measurement model estimated using WLSMV for ordinal indicators. Values associated with directional paths correspond to standardized factor loadings, whereas curved bidirectional paths represent standardized correlations among latent factors. All retained factor loadings were statistically significant at *p* < 0.001. Residual variances and item thresholds are omitted for visual clarity.

Although the final specification improved substantially over the initial model, fit was interpreted as reasonably acceptable rather than optimal. CFI exceeded.90 and RMSEA and SRMR were below.08, whereas TLI remained slightly below the conventional.90 reference value (Hair et al., 2019). No additional indicators were removed and no residual covariances were freely estimated solely to improve fit.

### 3.6 Reliability and convergent evidence

Composite Reliability (CR), Average Variance Extracted (AVE), and McDonald’s omega were examined for the refined 18-item configuration. CR was strong for Factor 1 (CR = 0.892), acceptable for Factor 2 (CR = 0.776), and borderline for Factor 3 (CR = 0.689). AVE values were 0.485, 0.369, and 0.427 for Factors 1–3, respectively (Table 8). None exceeded the conventional.50 reference value, indicating incomplete convergent validity, particularly for Factors 2 and 3 (Fornell and Larcker, 1981; Hair et al., 2019).

**TABLE 8:** Composite reliability and average variance extracted for the final CFA model.

Factor	Indicators	CR	AVE	Interpretation
Factor 1	9	0.892	0.485	Strong CR; AVE close to 0.50
Factor 2	6	0.776	0.369	Acceptable CR; limited convergent validity
Factor 3	3	0.689	0.427	Borderline CR; limited convergent validity

CR, composite reliability; AVE, average variance extracted. Evidence of convergent validity was interpreted jointly from AVE, standardized factor loadings, and reliability estimates rather than from any single index (Fornell and Larcker, 1981; Hair et al., 2019).

McDonald’s omega showed the same pattern, with ω = 0.880 for Factor 1, 0.755 for Factor 2, and 0.654 for Factor 3, indicating stronger score consistency for the first two factors and more limited reliability for the three-item third factor.

### 3.7 Discriminant differentiation

Discriminant differentiation was assessed using the Heterotrait–Monotrait ratio of correlations (HTMT). The coefficients were 0.182 between Factors 1 and 2, 0.301 between Factors 1 and 3, and 0.194 between Factors 2 and 3 (Table 9). All were well below the commonly applied 0.85 and 0.90 thresholds, supporting empirical differentiation among the retained factors (Henseler et al., 2015).

**TABLE 9:** Heterotrait–Monotrait ratios for the final three-factor model

Factor	F1	F2	F3
F1	1.000		
F2	0.182	1.000	
F3	0.301	0.194	1.000

Overall, the 18-item, three-factor configuration provided a substantially more parsimonious and better-fitting representation of the observed responses than the initial 23-item model. Nevertheless, Factor 3 remained comparatively weak, AVE remained below 0.50 across all factors, and the model resulted from sequential refinement within the same sample. The configuration should therefore be regarded as a provisional MDPAS-derived candidate structure requiring independent replication before stronger claims regarding structural stability, reliability, or broader applicability can be made.

## 4 Discussion

The present study examined the psychometric performance and cross-cultural applicability of the Motivational and Demotivational Patterns in Academic Settings (MDPAS) scale in a sample of Chilean university students. The findings provide partial support for the measurement structure proposed by Zeynali et al. (2019). Although the 36-item response matrix showed clear multidimensionality, the original five-dimensional organization was not uniformly recovered. Parallel analysis suggested up to six potentially retainable factors, but evidence for the sixth was marginal and the corresponding exploratory factor lacked adequate empirical definition. A theoretically informed five-factor solution subsequently showed that only three factors were sufficiently defined for model-based evaluation. Refinement of the resulting 23-item configuration yielded an 18-item, three-factor candidate model with substantially improved fit. However, incomplete convergent validity, comparatively weaker reliability and limited indicator coverage of Factor 3, and the absence of independent cross-validation require that this structure be regarded as provisional.

The factor-retention results illustrate the importance of integrating statistical and substantive criteria in psychometric evaluation. Kaiser’s eigenvalue-greater-than-one criterion produced an 11-factor solution, whereas parallel analysis suggested up to six factors. The sixth observed eigenvalue only marginally exceeded its random-data benchmark, and inspection of the corresponding six-factor solution showed that the additional factor was essentially defined by a single salient indicator. Thus, the statistical evidence indicated structural complexity but did not support interpretation of six stable latent dimensions. This finding is consistent with recommendations that factor retention should consider loading patterns, communalities, factor overdetermination, and substantive interpretability rather than rely on a single numerical criterion (Costello and Osborne, 2005; Goretzko, 2025; Lloret-Segura et al., 2014).

The theoretically informed five-factor solution provided only partial correspondence with the original MDPAS structure (Zeynali et al., 2019). Factors 1 and 2 contained the largest numbers of salient indicators, while Factor 3 formed a smaller but comparatively coherent cluster. In contrast, Factors 4 and 5 were insufficiently defined by unique indicators, and ten items failed to reach the prespecified salient-loading criterion of |λ|≥.40. Although the solution accounted for 36.77% of the extracted common variance, the interpretation of dimensionality depended more strongly on the loading structure and factorial definition than on variance explained alone (Floyd and Widaman, 1995).

The retained factors also showed substantial redistribution of indicators relative to the original MDPAS. Factor 1 retained a recognizable core of intrinsic and personally endorsed academic motivation but incorporated indicators from other original dimensions. Factor 2 combined collective/individual content with perfectionist and cooperative/competitive indicators, whereas Factor 3 integrated competitive and performance-oriented content that had originally been distributed across different dimensions. These patterns indicate incomplete structural transfer rather than direct replication of the original five-factor model.

From a cross-cultural psychometric perspective, this reorganization is noteworthy but should not be attributed exclusively to cultural differences. The study did not directly compare Chilean students with the original validation sample or test cross-cultural measurement invariance. Translation-related effects, sampling characteristics, disciplinary composition, institutional conditions, educational expectations, and genuine differences in motivational organization remain plausible explanations. The results therefore support caution regarding structural equivalence across contexts rather than evidence of a specifically Chilean motivational structure (Zeynali et al., 2019).

The exploratory reliability results were consistent with the factor-analytic evidence. Factor 1 showed good internal consistency, Factor 2 acceptable consistency, and Factor 3 borderline to moderate reliability, whereas Factors 4 and 5 were both weakly defined and unreliable. The overall Cronbach’s alpha of approximately 0.81 for the 36-item scale should therefore be interpreted descriptively rather than as evidence that the original subscale structure was reproduced. In multidimensional instruments, aggregate reliability can obscure substantial differences in score consistency across dimensions.

The initial 23-item, three-factor CFA showed inadequate fit, indicating that interpretable exploratory clusters did not automatically translate into an adequate measurement model. Sequential sensitivity analyses identified weak or ambiguous indicators and produced progressive improvements in fit. The final 18-item configuration reached χ^2^(132) = 415.586, *p* < 0.001, CFI = 0.911, TLI = 0.897, RMSEA = 0.078, and SRMR = 0.079, with standardized factor loadings ranging from 0.538 to 0.864. These values represent a substantial improvement over the initial model, although the fit is more appropriately characterized as reasonably acceptable than optimal, particularly because TLI remained slightly below 0.90 (Hair et al., 2019).

The refinement process was guided by converging evidence from standardized loadings, exploratory cross-factor patterns, and localized model diagnostics rather than by global fit indices alone. Nevertheless, all exploratory and confirmatory analyses were performed in the same sample. Consequently, the improved fit of the 18-item model demonstrates a better representation of the present data but does not constitute independent confirmation of the structure.

Construct-related evidence further illustrates the mixed psychometric performance of the candidate model. Composite Reliability was strong for Factor 1 (CR = 0.892), acceptable for Factor 2 (CR = 0.776), and borderline for Factor 3 (CR = 0.689). McDonald’s omega showed a similar pattern (ω = 0.880, 0.755, and 0.654, respectively). Factor 3 therefore remains the least stable dimension and is additionally limited by its three-indicator composition.

Convergent validity was less satisfactory. AVE values were 0.485 for Factor 1, 0.369 for Factor 2, and 0.427 for Factor 3, with none reaching the conventional 0.50 reference value. Although Factor 1 approached this criterion, the results indicate incomplete convergent validity overall, particularly for Factors 2 and 3. Acceptable CR or omega estimates therefore should not be interpreted as compensating automatically for insufficient AVE (Fornell and Larcker, 1981; Hair et al., 2019).

By contrast, discriminant differentiation was comparatively strong. HTMT values ranged from 0.182 to 0.301, substantially below commonly used thresholds of 0.85 and 0.90 (Henseler et al., 2015), and latent-factor correlations were similarly modest. These findings support empirical differentiation among the three retained dimensions. However, discriminant differentiation represents only one component of construct-related evidence and does not offset the limitations in convergent validity or Factor 3 reliability.

The exploratory analyses involving external variables provided additional information regarding the behavior of the composite factor scores derived from the 23-item pre-refinement configuration. Academic performance was positively associated with all three factors, although correlations were small. Age was positively associated with Factors 1 and 2, whereas academic semester was negatively associated with Factors 2 and 3. Failed courses were unrelated to all three dimensions. Because these analyses were based on the pre-refinement configuration, they should not be interpreted as external validation of the final 18-item candidate model.

The association between academic performance and motivational scores was modest, with the largest coefficient observed for Factor 1 (ρ = 0.281). These effects indicate limited shared variation rather than strong correspondence between motivation and academic performance. Moreover, the cross-sectional design does not permit conclusions regarding directionality. Motivational patterns may influence academic performance, academic success may reinforce particular motivational orientations, or both may reflect other unmeasured student or contextual characteristics.

Gender differences were similarly selective. Female students showed higher ranks on Factor 1, whereas male students showed higher ranks on Factor 3; Factor 2 showed no significant gender difference. The associated effect sizes were small, and the findings therefore do not support broad claims regarding gender-specific motivational profiles. Rather, they identify limited group differences that require replication before stronger theoretical interpretations are warranted.

Academic-area comparisons also revealed selective differences. Factor 1 differed between Engineering and Technology and Administration, Factor 3 between Engineering and Technology and Health Sciences, and Factor 2 showed the clearest differentiation, with Administration differing from the other academic areas. These results suggest that motivational patterns may vary across disciplinary environments, but the mechanisms underlying these differences cannot be established from the present data. Instructional climate, assessment practices, curriculum structure, peer competition, disciplinary norms, and perceived career opportunities were not directly measured. Because multiplicity adjustment was applied only to *post hoc* pairwise comparisons, these external-variable findings should remain exploratory.

The observed reorganization of the MDPAS can also be considered in relation to broader motivational theory. Self-Determination Theory distinguishes qualitatively different forms of regulation according to the extent to which behavior is autonomous or controlled, rather than treating intrinsic and extrinsic motivation as simple opposites (Ryan and Deci, 2020). From this perspective, the redistribution of MDPAS items may be compatible with the coexistence of personally endorsed motives, external pressures, achievement expectations, social influences, and demotivational experiences within students. Meta-analytic and profile-oriented research similarly indicates that multiple motivational regulations can coexist within students and relate differently to persistence, academic performance, and well-being (Howard et al., 2021; Litalien et al., 2024). Research grounded in SDT further highlights the role of perceived competence, psychological need satisfaction, and autonomy-supportive educational conditions in shaping the quality of student motivation (Bureau et al., 2022; Guay, 2022).

The competitive and performance-oriented content of Factor 3 may also be interpreted in relation to perfectionism research. High personal standards can reflect adaptive striving in some contexts but maladaptive concerns, excessive self-evaluation, or externally pressured achievement in others (Cowie et al., 2018; Ghosh and Roy, 2017). The clustering of competitive and performance-related indicators may therefore reflect a broader motivational configuration in which achievement striving and social comparison are not fully separable. However, the present psychometric design cannot establish the psychological mechanisms underlying this pattern, and such interpretations should be treated as hypotheses for future research.

Several limitations qualify the findings. First, participants were recruited through convenience sampling from a single public university in northern Chile, limiting generalizability to other institutions, regions, and higher education systems. Although the sample included multiple academic areas, this heterogeneity does not substitute for multi-institutional sampling.

Second, the cross-sectional and non-experimental design precludes causal interpretation of associations with academic and sociodemographic variables. Longitudinal studies are needed to determine whether motivational patterns change across university trajectories or predict subsequent academic outcomes.

Third, the adaptation process included translation, expert review, and pilot testing but did not include formal back-translation. Full linguistic equivalence with the original instrument therefore cannot be established. Future studies should incorporate independent back-translation and explicit assessment of semantic, conceptual, and functional equivalence.

A central limitation concerns the use of the same sample for EFA, CFA, and model refinement. The resulting 18-item configuration may therefore partly reflect sample-specific characteristics. Independent replication should evaluate this model using a prespecified CFA without further exploratory modification. Subsequent studies should also examine measurement invariance across gender, academic disciplines, institutions, geographical regions, and stages of university progression, including configural, loading, threshold/scalar, and latent-mean equivalence where sample size permits.

The reduction from the original 36-item, five-dimensional MDPAS to an 18-item, three-factor configuration also has conceptual implications. The candidate model retains only part of the content represented in the original instrument and should not be interpreted as a directly interchangeable abbreviated version of the five-subscale MDPAS. The three empirical factors do not map perfectly onto the original dimensions proposed by Zeynali et al. (2019), and scores derived from the candidate configuration should therefore not be assumed to represent identical constructs.

This distinction also limits current applied use. The candidate configuration may serve as a research framework for examining group-level motivational and demotivational patterns, but the available evidence does not support diagnostic use at the individual level or application to high-stakes educational decisions. Such uses would require substantially stronger evidence regarding structural stability, temporal reliability, convergent and criterion-related validity, measurement invariance, normative interpretation, and predictive utility.

Overall, the study provides evidence that the MDPAS captures meaningful multidimensional variation in Chilean university students while showing that its original five-dimensional structure does not transfer uniformly to this sample. The refined 18-item, three-factor configuration offers a more parsimonious representation of the observed responses, with improved global fit and clear discriminant differentiation. However, incomplete convergent validity, comparatively weaker reliability and limited indicator coverage of Factor 3, within-sample refinement, and the absence of independent confirmation constrain the strength of the validation claims. The candidate structure therefore warrants further investigation through independent replication and broader cross-cultural psychometric evaluation.

## 5 Conclusion

This study provides partial psychometric support for the Motivational and Demotivational Patterns in Academic Settings (MDPAS) scale in a Chilean higher education context. The original five-dimensional structure was not uniformly recovered, and only three factors showed sufficient empirical definition to support subsequent model-based evaluation. Refinement of the exploratory 23-item configuration yielded an 18-item, three-factor candidate model with substantially improved fit. However, the evidence remains provisional rather than confirmatory.

The refined model showed statistically significant factor loadings and clear discriminant differentiation according to HTMT criteria. Reliability was comparatively strong for Factor 1 and acceptable for Factor 2, whereas Factor 3 remained less robust and was represented by only three indicators. In addition, AVE remained below the conventional.50 reference value across all factors, indicating incomplete convergent validity. The resulting configuration should therefore be interpreted as a candidate MDPAS-derived structure rather than as a definitively validated shortened version of the original instrument.

The study also showed that cross-context application of the MDPAS should not assume structural equivalence with the original five-factor model proposed by Zeynali et al. (2019). The redistribution and weak performance of several indicators suggest that the dimensional organization of the instrument may vary across educational contexts. However, because no direct cross-cultural comparison or measurement-invariance analysis was conducted, these differences cannot be attributed specifically to Chilean culture.

Associations with sociodemographic and academic variables were selective and generally small. Because these analyses were based on the 23-item pre-refinement configuration, they should be interpreted as exploratory relationships rather than as external validation of the final 18-item model. The cross-sectional design further precludes causal interpretation.

Several limitations constrain the strength of the conclusions. Participants were recruited through convenience sampling from a single public university, the adaptation did not include formal back-translation, and the EFA, CFA, and model refinement were conducted in the same sample. These features limit generalizability, linguistic equivalence, and independent confirmation of the refined structure.

Future research should therefore prioritize independent replication of the 18-item, three-factor candidate model in broader Chilean and Latin American samples. Subsequent studies should evaluate measurement invariance across gender, academic disciplines, institutions, geographical contexts, and stages of university progression, as well as temporal stability and associations with established motivational and academic outcomes.

At its current stage of development, the candidate configuration may be useful for research on group-level motivational and demotivational patterns in higher education. However, the available evidence does not support diagnostic or high-stakes individual use. Stronger evidence regarding structural stability, reliability, convergent and criterion-related validity, measurement invariance, and predictive utility is required before broader applied use can be recommended.

Overall, the findings extend the cross-context psychometric evaluation of the MDPAS while showing that its original five-dimensional organization is not fully reproduced in this Chilean university sample. The 18-item, three-factor configuration represents a more parsimonious candidate structure, but independent replication remains necessary before it can be regarded as stable or broadly applicable.

## Data Availability

The raw data supporting the conclusions of this article will be made available by the authors, without undue reservation.
